# Individualized fluid administration for critically ill patients with sepsis with an interpretable dynamic treatment regimen model

**DOI:** 10.1038/s41598-020-74906-z

**Published:** 2020-10-21

**Authors:** Zhongheng Zhang, Bin Zheng, Nan Liu

**Affiliations:** 1 Department of Emergency Medicine, Sir Run Run Shaw Hospital, Zhejiang University School of Medicine, No 3, East Qingchun Road, Zhejiang Province Hangzhou, 310016 China; 2 Department of Surgery, 2D, Walter C Mackenzie Health Sciences Centre, University of Alberta, Edmonton, AB 2 Canada; 3 Duke-NUS Medical School, National University of Singapore, Singapore, Singapore; 4 Health Services Research Centre, Singapore Health Services, Singapore, Singapore

**Keywords:** Bacterial infection, Prognosis

## Abstract

Fluid strategy is the key to the successful management of patients with sepsis. However, previous studies failed to consider individualized treatment strategy, and clinical trials typically included patients with sepsis as a homogeneous study population. We aimed to develop sequential decision rules for managing fluid intake in patients with sepsis by using the dynamic treatment regimen (DTR) model. A retrospective analysis of the eICU Collaborative Research Database comprising highly granular data collected from 335 units at 208 hospitals was performed. The DTR model used a backward induction algorithm to estimate the sequence of optimal rules. 22,868 patients who had sepsis according to the Acute Physiology and Chronic Health Evaluation (APACHE) IV diagnosis group were included. Optimal fluid management (liberal [> 40 ml/kg/d] versus restricted [< 40 ml/kg/d]) strategy were developed on the Day 1, 3 and 5 after ICU admission according to current states and treatment history. Important determinants of optimal fluid strategy included mean blood pressure, heart rate, previous urine output, previous fluid strategy, ICU type and mechanical ventilation. Different functional forms such as quadratic function and interaction terms were used at different stages. The proportion of subjects being inappropriately treated with liberal fluid strategy (i.e. those actually received liberal fluid strategy, but could have longer survival time if they received restricted fluid strategy) increased from day 1 to 5 (19.3% to 29.5%). The survival time could be significantly prolonged had all patients been treated with optimal fluid strategy (5.7 [2.0, 5.9] vs. 4.1 [2.0, 5.0] days; p < 0.001). With a large volume of sepsis data, we successfully computed out a sequence of dynamic fluid management strategy for sepsis patients over the first 5 days after ICU admission. The decision rules generated by the DTR model predicted a longer survival time compared to the true observed strategy, which sheds light for improving patient outcome with the aim from computer-assisted algorithm.

## Introduction

Management of critically ill patients with sepsis is imperative as sepsis can attack all human organs leading to a fatal consequence in a short period of time. Among multiple aspects for saving the life of patients with sepsis, we believe that fluid management strategy remains essential to the successful treatment because the cardiac and renal functions, which play important roles in the physiological regulation of fluid balance, are usually impaired in sepsis. Numerous studies have been conducted to explore an optimal fluid strategy. A trio of multinational trials named Protocolized Care for Early Septic Shock (ProCESS), Australasian Resuscitation in Sepsis Evaluation (ARISE), and Protocolized Management in Sepsis (ProMISe) aimed to investigate whether early goal directed therapy (EGDT) could improve the mortality outcome^1–3^. The results showed that EGDT did not significantly reduce mortality rate as compared with the usual care group. Thus, the optimal fluid strategy remains largely unknown. One important limitation of these trials is that they included all sepsis patients without considering between-subject heterogeneity. As a matter of fact, there has been large body of evidence showing that sepsis is highly heterogenous and different subphenotypes can have distinct responses to fluid administration^4^. Latent profile analysis and hierarchical clustering are two major techniques to identify subphenotypes of sepsis^5^, which however are limited by two factors. First, the capacity to establish a causal relationship between treatment and subphenotypes is insufficient. Second, most studies used cross-sectional data when modeling the subphenotypes. We should aware that fluid management in a sepsis patient is a dynamic process over entire treatment course; we should not solely focus on the initial fluid strategy but ignore the importance at later stages. There was evidence that de-resuscitation (negative fluid balance) at later phase of sepsis is beneficial^6^. Therefore, we need to adjust our fluid management strategy according to the patient’s changing state and treatment history.

Reinforcement learning (RL) is a well-developed algorithm of machine learning focusing on how a computing system gives a response based on environmental inputs so the system can maximize cumulative reward^7^. RL has been used to determine the optimal fluid treatment strategy in sepsis^8–10^. However, these results are difficult to interpret due to the black-box algorithms, which significantly prohibited their use in clinical practice. Dynamic treatment regimen (DTR) method borrows the idea of RL, but simplifies the functional forms of the multi-dimensional feature space, which can help clinicians to better understand the decision rules ^11^. This study aimed to optimize fluid treatment strategy (liberal versus restrictive) by using DTR during the course of sepsis. Instead of using cross-sectional data on one single phase, we applied DTR model on three different days (Day 1, Day 3, and Day 5) over the first week of treatment with a purpose of examine dynamic nature of the fluid management on sepsis treatment. We hypothesized that the optimal fluid treatment strategy recommended by DTR would be better than the treatment actually received in terms of survival outcome.

## Methods

### Database and study population

The present study utilized the eICU Collaborative Research Database, which is a multi-center intensive care unit (ICU) database with high granularity data for over 200,000 admissions to ICUs monitored by eICU Programs across the United States^12^. Patients with sepsis were included for our analysis. Sepsis was defined according to the sepsis-2.0 definition^13^. Sepsis-2.0 was defined when a patient had suspected or documented infection plus 2 of the SIRS criteria including temperature $\le$ 36℃ or $\ge$ 38℃,heart rate $\ge$ 90 bpm, respiratory rate $\ge$ 20 breath/min or PaCO2 < 32 mmHg, and white blood cell count > 12,000 or < 4000 cells/mm^3^ or > 10% band. Sepsis was further categorized by infection sites including cutaneous, gastrointestinal, pulmonary, urinary tract, other location and unknown location^14^.

### Some definitions

The primary outcome was patient status (alive versus expired) at hospital discharge, which was considered as time to event survival data. Subjects who were discharged alive were considered as censored. We further defined the survival time of the patient from ICU admission to expiration. Patients who discharged alive were considered as censored. The state (feature space) of patients at each stage (Day 1, 3 and 5 after ICU admission) was constructed by variables including heart rate (HR), mean blood pressure (mBP), respiratory rate (RR), Glasgow coma scale (GCS), body temperature, creatinine, lactate, hemoglobin, bilirubin, use of vasopressor, platelet count, PaO_2_/FiO_2_ (P/F) ratio, daily urine output and the use of mechanical ventilation (MV).

Total fluid intake was calculated as the sum of fluid intake for a 24-h interval. We assumed that the fluid strategy was determined at the beginning of each interval. Fluid intake was also normalized by the body weight. We defined liberal and restricted fluid administration as ≥ 40 ml/kg/day and < 40 ml/kg/day, respectively. This cutoff point was chosen according to the distribution of the fluid intake in the study population so that both categories would have balanced number of observations.

### Statistical analysis

Descriptive statistics were analyzed conventionally using the *CBCgrps* package in R^15^.

The DTR model estimated a sequence of treatment strategy that maximized the survival time across stages of clinical intervention^16^. We defined three stages on day 1, 3 and 5 after ICU entry and recorded the survival times within each stage. For the first stage, the survival time ${T}_{1}$ corresponded to the time (in days) from the beginning of day 1 to day 3 or day 1 to death if the patient died before day 3. The survival times ${T}_{2}$ and ${T}_{3}$ were defined similarly.

The DTR model used a backward induction algorithm to estimate the sequence of optimal rules. In the first step, the optimal stage 3 decision rule (day 5) was estimated by modeling the counterfactual survival time in stage 3 (${T}_{3}^{{a}_{1},{a}_{2},{a}_{3}}$) as a function of the treatment received on day 5 (restricted [${a}_{3}=0$] or liberal [${a}_{3}=1$] fluid administration) and of the feature variables measured on day 5 or before (${h}_{3\beta }$ and ${h}_{3\psi }$):

$$\mathrm{log}\left({T}_{3}^{{a}_{1},{a}_{2},{a}_{3}}\right)={\beta }_{3}^{T}{h}_{3\beta }+{a}_{3}{\psi }_{3}^{T}{h}_{3\psi }+{\epsilon }_{3}$$

The term ${a}_{3}{\psi }_{3}^{T}{h}_{3\psi }$ is the stage 3 blip function. It represents the effect of receiving liberal fluid administration instead of restricted fluid administration and its interaction with feature variables. The term ${\psi }_{3}$ is a vector of coefficients for feature variables and ${h}_{3\psi }$ represents information (previous treatment, covariates and survival times) available prior to making the stage 3 treatment. We included age, mBP, unit type, HR, RR, use of vasopressor, MV, urine output, temperature, P/F ratio, and treatment strategies on days 3 and 4 as potential features to determine the optimal treatment. Variables were excluded from the final model if the statistical significance level was greater than 0.05. We retained some important variables such as age and mBP according to expertise. The optimal stage 3 treatment was identified for each subject who entered stage 3 by ${a}_{3}^{opt}=\mathrm{I}({\psi }_{3}^{T}{h}_{3\psi }>0)$. If optimal rule recommends liberal fluid administration on day 5 if the condition ${\psi }_{3}^{T}{h}_{3\psi }>0$ is satisfied, and restricted fluid administration otherwise.

In the second step, we estimated the optimal stage 2 treatment strategy by modeling the counterfactual survival time ${\stackrel{\sim }{T}}^{{a}_{1},{a}_{2},{a}_{3}^{opt}}={T}_{2}+{T}_{3}\times \mathrm{exp}({\psi }_{3}^{T}{h}_{3\psi }[{a}_{3}^{opt}-{a}_{3}]))$. It represents the survival time from day 3 (stage 2) onwards had the patient received his optimal stage 3 treatment. It is equal to the observed survival time ${T}_{2}+{T}_{3}$ if the patient received his optimal stage 3 treatment and is larger that ${T}_{2}+{T}_{3}$ otherwise. A similar strategy as in the third stage was adopted to find the optimal stage 2 treatment rule ${a}_{2}^{opt}=\mathrm{I}({\psi }_{2}^{T}{h}_{2\psi }>0)$.

In the third step, we proceeded to the optimization of the first stage treatment by modeling the counterfactual survival time ${\stackrel{\sim }{T}}^{{a}_{1},{a}_{2}^{opt},{a}_{3}^{opt}}$ (overall survival time had both the stage 2 and 3 treatments been optimal). The optimal stage 1 treatment rule was also of the form ${a}_{1}^{opt}=\mathrm{I}({\psi }_{1}^{T}{h}_{1\psi }>0)$. The DTR model was built with the DTRreg package (v1.5) in R ^17^.

### Ethics approval and consent to participate

Data were available on request. This study was an analysis of the third-party anonymized databases with pre-existing IRB approval.

## Results

### Baseline characteristics of included subjects

**Figure 1:**
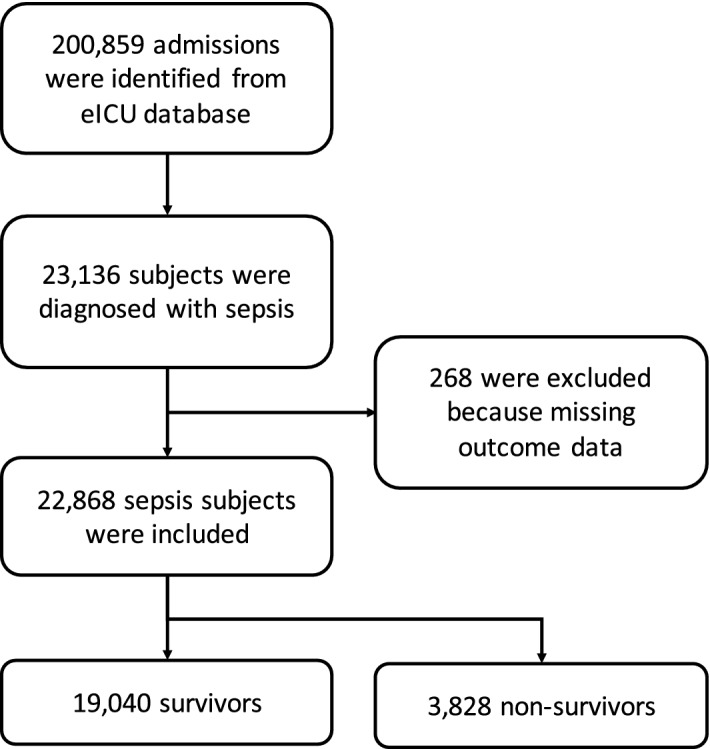
Flowchart of patient selection.

A total of 22,868 patients with sepsis were identified from the database (Fig. 1), comprising 19,040 survivors and 3,838 non-survivors at hospital discharge (Table 1). There was no significant difference between the two groups in sex, ethnicity, admission height and presence of AIDS. Survivors were significantly younger (median [IQR]: 65 [53, 77] vs. 72 [60,83] years; p < 0.001), had larger weight (77.7 [64.1, 96.2] vs. 74.3 [61, 90.9] kg; p < 0.001), higher mBP (58 [50, 68] vs. 51 [40, 60] mmHg; p < 0.001), and more likely to have renal/UTI (24% vs. 15%; p < 0.001) than the non-survivors (Table 2).

**Table 1:** Baseline demographic characteristics between survivors and non-survivors.

Variables	Total (n = 22,868)	Alive(n = 19,040)	Expired(n = 3828)	p
Gender, Male, No. (%)	11,600(51)	9,653(51)	1,947(51)	0.898
Age	66(54,78)	65(53,77)	72(60,83)	< 0.001
**Ethnicity, No. (%)**				0.91
Missing	200(1)	165(1)	35(1)	
African American	2282(10)	1887(10)	395(10)	
Asian	438(2)	365(2)	73(2)	
Caucasian	17,714(77)	14,760(78)	2954(77)	
Hispanic	920(4)	757(4)	163(4)	
Native American	201(1)	170(1)	31(1)	
Other/Unknown	1113(5)	936(5)	177(5)	
Admission height	167.75(160,177.8)	168(160,177.8)	167.6(160,177.8)	0.194
Admission weight	77.1(63.5,95.3)	77.7(64.1,96.2)	74.3(61,90.9)	< 0.001
**Admit source, No. (%)**				< 0.001
Operating Room	46(0)	42(0)	4(0)	
Recovery Room	31(0)	28(0)	3(0)	
Chest Pain Center	3(0)	3(0)	0(0)	
Floor	4564(21)	3530(20)	1034(28)	
Other ICU	127(1)	98(1)	29(1)	
Other Hospital	481(2)	373(2)	108(3)	
Direct Admit	1291(6)	1058(6)	233(6)	
Emergency Department	15,158(70)	12,910(72)	2248(61)	
**Unit type, No. (%)**				< 0.001
CCU-CTICU	1339(6)	1119(6)	220(6)	
CSICU	379(2)	312(2)	67(2)	
CTICU	162(1)	126(1)	36(1)	
Cardiac ICU	1347(6)	1094(6)	253(7)	
MICU	3068(13)	2475(13)	593(15)	
Med-Surg ICU	15,270(67)	12,836(67)	2434(64)	
Neuro ICU	433(2)	346(2)	87(2)	
SICU	870(4)	732(4)	138(4)	

Characteristics expressed as median [IQR] unless specified otherwise.

Abbreviations: IQR: interquartile range; CCU-CTICU: coronary care unit- cardiothoracic ICU; CSICU: cardiac surgery ICU; CTICU: cardiothoracic ICU; SICU: surgical ICU; MICU: medical ICU; GI: gastrointestinal; BUN: blood urea nitrogen.

**Table 2:** Clinical characteristics of included patients.

Variables	Total (n = 22,868)	Alive(n = 19,040)	Expired(n = 3,828)	p
**Infection sites, No. (%)**				< 0.001
GI	2843(12)	2264(12)	579(15)	
Cutaneous/soft tissue	1903(8)	1684(9)	219(6)	
Gynecologic	75(0)	65(0)	10(0)	
Other	1500(7)	1192(6)	308(8)	
Pulmonary	8751(38)	7110(37)	1641(43)	
Renal/UTI (including bladder)	5212(23)	4621(24)	591(15)	
unknown	2584(11)	2104(11)	480(13)	
MV, No. (%)	5448(24)	3807(20)	1641(43)	< 0.001
GCS	13(9.81,15)	13.72(10.12,15)	10(7,13.8)	< 0.001
Bilirubin (mg/dl), median (IQR)	1.23(0.59,3.2)	1.2(0.5,3.08)	1.6(0.7,3.8)	< 0.001
Creatinine (mg/dl), median (IQR)	1.37(0.86,2.43)	1.28(0.82,2.25)	1.97(1.2,3.12)	< 0.001
Platelet ($\times {10}^{9}/L$), median (IQR)	178(119,248)	181(126,250)	157(85,234)	< 0.001
PaO2 (mmHg), median (IQR)	85(63.26,115.57)	86.33(64,116.28)	79(60.36,110.94)	< 0.001
MBP (mmHg), median (IQR)	57(49,67)	58(50,68)	51(40,60)	< 0.001
SOFA, median (IQR)	8(6,10)	7(6,9)	10(8,12)	< 0.001
Urine Output (ml/d), median (IQR)	50(0,1000)	100(0,1100)	0(0,575)	< 0.001
Temperature (℃), median (IQR)	37.3(36.9,38.11)	37.33(36.9,38.11)	37.3(36.8,38.2)	< 0.001
RR (/min), median (IQR)	29(24,35)	28(24,34)	32(26,38)	< 0.001
HR (/min), median (IQR)	109(95,124.99)	108(94,123)	117(100.36,134)	< 0.001
pH, median (IQR)	7.35(7.27,7.41)	7.36(7.29,7.42)	7.3(7.2,7.38)	< 0.001
Hematocrit (%), median (IQR)	30.6(26.4,35)	30.8(26.7,35.1)	29.3(25.1,34.25)	< 0.001
Albumin (mg/dl), median (IQR)	2.5(2.1,2.9)	2.5(2.1,2.9)	2.3(1.9,2.71)	< 0.001
BUN (mg/dl), median (IQR)	28(17,46)	26(16,43)	40(26,59)	< 0.001
Glucose (mg/dl), median (IQR)	132(93,206)	132(94,204)	134(86,215)	0.011
**Comorbidities, No. (%)**				
AIDS	66(0)	50(0)	16(0)	0.152
Hepatic failure	463(2)	320(2)	143(4)	< 0.001
lymphoma	211(1)	160(1)	51(1)	0.006
Metastatic cancer	748(3)	530(3)	218(6)	< 0.001
leukemia	338(2)	240(1)	98(3)	< 0.001
immunosuppression	1232(6)	947(5)	285(8)	< 0.001
Cirrhosis	611(3)	416(2)	195(5)	< 0.001

Characteristics expressed as median [IQR] unless specified otherwise.

Abbreviations: AIDS: acquired immune deficiency syndrome; IQR: interquartile range; SOFA: sequential organ failure assessment; MBP: mean blood pressure; RR: respiratory rate; GCS: Glasgow coma scale; MV: mechanical ventilation.

### Fluid intake

Survivors consistently received less fluid intake from day 1 to 10 than non-survivors (day 1: 38.07 [17.71, 68.96] vs. 43.93 [17.17, 83.97] ml/kg/day; p < 0.001). The significance level of 0.05 was not reached on day 4, but the survivors still had less fluid intake than non-survivors (27.9 [12.85, 59.86] vs. 30.44 [13.88, 66.41] ml/kg/day; p = 0.074). The amount of fluid intake decreased gradually from day 1 to 10 from 38.88 (IQR: 17.63–71.52) to 27.88 (IQR: 14.12–56.71) ml/kg/day (Table 3). However, it was not straightforward to improve survival outcome by simply reducing fluid intake because the association was not causality. Thus, we needed to employ DTR to prescribe optimal amount of fluid for each individual, based on their current status and treatment history.

**Table 3:** Fluid intake per kilogram for the first 10 days after ICU entry.

Fluid intake (ml/kg/day)	Total (n = 22,868)	Alive(n = 19,040)	Expired(n = 3828)	p
Day 1	38.88(17.63,71.52)	38.07(17.71,68.96)	43.93(17.17,83.97)	< 0.001
Day 2	32.67(14.87,62.71)	31.93(14.69,60.65)	36.77(16.28,73.04)	< 0.001
Day 3	30(13.09,61.74)	29.49(12.99,60.72)	33(14.01,67.3)	0.025
Day 4	28.4(13.05,60.85)	27.9(12.85,59.86)	30.44(13.88,66.41)	0.074
Day 5	27.81(13.06,60.81)	27.09(12.77,58.75)	31.67(14.31,70.96)	0.005
Day 6	27.86(12.81,57.58)	26.35(12.38,53.09)	33.7(14.22,77.18)	< 0.001
Day 7	26.74(13.03,54.93)	26.35(12.9,53.09)	29.49(13.58,61.18)	0.043
Day 8	27.94(13.77,58.23)	26.32(13.39,54.84)	33.99(15.3,74.1)	0.001
Day 9	27.75(13.48,57.54)	26.86(13.33,53.74)	32.61(13.96,67.25)	0.021
Day 10	27.88(14.12,56.71)	27.06(13.47,51.67)	30.94(15.08,69.36)	0.022

Fluid intake expressed as median [IQR] unless specified otherwise.

### The DTR and its interpretation

The DTR model estimated a sequence of treatment rules to recommend liberal or restricted fluid intake across stages in order to obtain a better survival outcome. On day 1 (stage 1), subjects should receive liberal fluid administration if they satisfied the following condition:

$$-1.2478- 0.00669\mathrm{ age}+0.4129\mathrm{CSICU}+0.0775\mathrm{CTICU}+ 0.2146\mathrm{ Cardiac ICU }- 0.1976\mathrm{ MICU }- 0.0742\mathrm{ Med}-\mathrm{Surg ICU }+ 1.4847\mathrm{ Neuro ICU }+ 0.4396\mathrm{ SICU }+ 0.0853 \left( \frac{{\mathrm{HR}}_{1}}{20}\right)+ 0.7074 \left( \frac{{\mathrm{mBP}}_{1}}{20}\right)- 0.1139 {\left(\frac{{\mathrm{mBP}}_{1}}{20}\right)}^{2}+ 0.3567 {\mathrm{MV}}_{1}>0$$

Otherwise, they should receive restricted fluid administration in order to achieve a better survival outcome. Subscripts of variables in the equation denote the ICU days. Positive coefficient of a variable means that the presence or increase in the variable favors liberal fluid administration. The rule indicated that liberal fluid administration was more likely to be beneficial to patients from cardiac surgery ICU (CSICU) (coefficient: 0.413; 95% CI 0.370–0.455; p < 0.001), NeuroICU (1.485; 95% CI 1.438–1.532; p < 0.001) and surgical ICU (SICU) (0.44; 95% CI 0.357–0.522; p < 0.001); but was harmful to patients from medical ICU (MICU) (− 0.198 [− 0.307, − 0.088]; p = 0.001), Med-Surg ICU (− 0.074 [− 0.136, − 0.013]; p = 0.048). Higher HR mandated more fluid administration (0.085 [0.056, 0.115]; p < 0.001). Patients on MV also required more fluid intake (0.357 [0.066, 0.648]; p = 0.045). The impact of mBP was a parabola opens down with the line of symmetry at mBP = 62.1 mmHg. Clinical interpretation was that more fluid intake was warranted for patients with mBP < 62.1 mmHg; while more restricted fluid administration was preferred with increasing mBP if mBP > 62.1 mmHg.

The condition for liberal fluid administration on day 3 (stage 2) was:

$$0.1160-0.0037 {\mathrm{Temper}}_{3}- 0.1630 \left(\frac{{\mathrm{mBP}}_{3}}{20}\right)+ 0.1754\left( \frac{{\mathrm{urineOutput}}_{2}}{1000}\right)+ 0.0190 \left( \frac{{\mathrm{urineOutput}}_{1}}{1000}\right)+ 0.5338 {\mathrm{MV}}_{3}+ 0.1514 {\mathrm{LiberalFluid}}_{2}+ 0.2415 {\mathrm{LiberalFluid}}_{1}> 0$$

This rule indicated that more urine output (coefficient: 0.175; 95% CI: 0.128–0.223; p < 0.001 for day 2) in previous days mandated liberal fluid administration on day 3. The mBP on day 3 did not follow a parabolic function (the quadratic term is not statistically significant). In this case, higher mBP mandated less fluid administration.

The condition for liberal fluid administration on day 5 (stage 3) was:

$$-0.5573+0.6556 {\mathrm{MV}}_{5}+ 0.0017 {\mathrm{HR}}_{5}- 0.0019 {\mathrm{mBP}}_{5}+ 0.1147 {\mathrm{LiberalFluid}}_{3}+ 0.4317 {\mathrm{LiberalFluid}}_{4}- 0.4571 {\mathrm{LiberalFluid}}_{3}\bullet {\mathrm{LiberalFluid}}_{4}> 0$$

There was a significant interaction (coefficient: − 0.457; 95% CI − 0.873 to − 0.041; p = 0.039) between day 3 and day 4 treatment strategy for determining the day 5 treatment strategy. The interaction indicated that if liberal fluid administration was given on day 3, restricted fluid administration was more likely to be beneficial on day 5 if liberal fluid balance was also given on day 4 (Table 4).

**Table 4:** Coefficients for the blip functions.

	Coefficient (95% CI)	P value
**Blip function on day 1**		
Intercept	− 1.248(− 2.5,0.004)	0.118
Age (per 10-year increase)	− 0.067(− 0.163,0.029)	0.314
**Unit type (CCU-CTICU as reference)**		
CSICU	0.413(0.37,0.455)	< 0.001
CTICU	0.078(− 0.072,0.228)	0.477
Cardiac ICU	0.215(0.184,0.245)	< 0.001
MICU	− 0.198(− 0.307,− 0.088)	0.001
Med-SurgICU	− 0.074(− 0.136,− 0.013)	0.048
Neuro ICU	1.485(1.438,1.532)	< 0.001
SICU	0.44(0.357,0.522)	< 0.001
HR_1_ (per 20-beat increase)	0.085(0.056,0.115)	< 0.001
MBP_1_ (per 20-mmHg increase)	0.707(0.227,1.188)	0.012
${\mathrm{MBP}}_{1}^{2}$	− 0.114(− 0.215,− 0.013)	0.041
MV_1_	0.357(0.066,0.648)	0.045
**Blip function on day 3**		
Intercept	0.116(− 0.249,0.481)	0.657
Temperature_3_	− 0.004(− 0.006,− 0.002)	< 0.001
MBP_3_	− 0.163(− 0.281,− 0.045)	0.021
Urine output_2_	0.175(0.128,0.223)	< 0.001
Urine output_1_	0.019(− 0.02,0.058)	0.509
MV_3_	0.534(0.396,0.671)	< 0.001
Fluid strategy_2_	0.151(0.11,0.193)	< 0.001
Fluid strategy_1_	0.241(0.213,0.27)	< 0.001
**Blip function on day 5**		
Intercept	− 0.557(− 0.807,− 0.308)	< 0.001
MV_5_	0.656(0.6,0.711)	< 0.001
HR_5_	0.002(0.001,0.002)	< 0.001
MBP_5_	− 0.002(− 0.008,0.004)	0.64
Fluid strategy_3_	0.115(− 0.136,0.366)	0.535
Fluid strategy_4_	0.432(0.198,0.666)	0.001
${\mathrm{Fluid strategy}}_{3}\times {\mathrm{Fluid strategy}}_{4}$	− 0.457(− 0.873,− 0.041)	0.039

Abbreviations: MBP: mean blood pressure; HR: heart rate; MV: mechanical ventilation; CCU-CTICU: coronary care unit- cardiothoracic ICU; CSICU: cardiac surgery ICU; CTICU: cardiothoracic ICU; SICU: surgical ICU; MICU: medical ICU.

Table 5 compares the difference between optimal and observed treatments for each subject. The proportion of patients who actually received liberal fluid administration but who would have better survival outcome had they received restricted fluid increased from 19.3% on day 1 to 29.5% on day 5. This result indicated that patients were more likely to receive too much fluid at latter phase of sepsis than that at the early phase. If all patients had received the optimal treatment strategy at all stages as recommended by DTR, the survival time could be significantly prolonged (5.7 [2.0, 5.9] vs. 4.1 [2.0, 5.0] days; p < 0.001).

**Table 5:** Cross table showing the difference between optimal and actually received treatment.

Actually-received treatment	Optimal treatment	
	Restricted fluid administration	Liberal fluid administration
**Day 1, No. (%)**		
Restricted fluid administration	10,728(47)	5,248(23)
Liberal fluid administration	4,410(19.3)	2,455(10.7)
**Day 3, No. (%)**		
Restricted fluid administration	6,994(57.8)	2,374(19.6)
Liberal fluid administration	1,737(14.4)	992(8.2)
**Day 5, No. (%)**		
Restricted fluid administration	2,549(43.1)	1,029(17.4)
Liberal fluid administration	1,746(29.5)	595(10.1)

The cross table shows the difference between the optimal treatment and the treatment that was received by the patient. For example, 10,728 patients received restricted fluid administration on day 1, which was consistent with the optimal treatment strategy. However, there were 4,410 patients who received liberal fluid administration, but they were expected to have better clinical outcome (survive to discharge) had they been treated with restricted fluid administration. In contrast, 5,248 patients received restricted fluid administration but would have had a better outcome had they received liberal fluid administration. Data on days 3 and 5 are interpreted in the same way.

## Discussion

This study developed a simple and interpretable algorithm for calculating fluid management strategy in critically ill patient with sepsis. The DTR model was modified from complex ML algorithm and taking patients' current characteristics and their treatment history into the calculation. The fluid management recommendation was report on the Day 1, 3, and 5 after ICU admission. We also showed that following the optimal treatment strategy at each stage significantly improved the survival time. In other words, our hypothesis was supported. Inspection over different stages, we observed discrepancy between calculated and actual fluid administration. Specifically, we found that sepsis patients were more likely to receive inappropriate liberal fluid administration at later stage than that at early stage (Table 4). Here, we will discuss why calculated fluid management strategy by the DTR model would predict a better clinical outcome, and why clinician tended to employ inappropriate (liberal) fluid administration at the later stage of sepsis.

Conventionally, fluid administration was guided by a variety of biomarkers reflecting the circulatory status such as serum lactate, ScvO2, and capillary refill time. The 2016 version of the surviving sepsis bundle recommended maintaining MAP > 65 mmHg and normalization of lactate^18^. However, most studies investigating the effectiveness of fluid resuscitation targeting these parameters showed neural effect on mortality^19–21^. For example, the ANDROMEDA-SHOCK trial investigated fluid strategy by targeting lactate clearance versus normalization of capillary refill time, which showed no difference between the two groups^22^. One important reason for the failure of these trials lied in the heterogeneity of the sepsis population. It has been shown that sepsis population was highly heterogenous and that it could be further categorized into subphenotypes based on routinely measured clinical characteristics^4,5,23^. It is important to give different fluid strategy to different patients at different stages^24^; however, it is almost impossible to fulfill this goal using conventional model based on physician's judgement over a few clinical variables. Aids from computing technology are needed for this assignment. RL is a novel technique to help an agent to select appropriate treatment to maximize final outcome based on current states. By feeding in highly granular electronic healthcare data, RL is capable to adopt clinical reasoning from experienced physicians and yield the optimal appropriate fluid management strategy based on current condition and the treatment history for each individual patient ^8,9^. However, the RL method based on deep learning algorithm is difficult to understand for ICU physician and cannot be easily implemented in clinical practice. Thus, we used a simplified RL algorithm by considering a binary variable space (liberal vs. restricted fluid administration) and modeling the decision rule (blip function) with generalized linear model. The resultant DTR model was clinically interpretable and could easily guide clinical practice, which was an improvement over other less accessible RL algorithms.

The appropriate selection of feature variables for the blip function was essential for applying the DTR model. Although the SSC guideline recommended targeting lactate clearance as the resuscitation guide, lactate was not statistically significant in the blip function, and thus we excluded this variable. CVP was not included in the DTR model in our study because it has been documented less useful for assessing fluid status^25^. The mBP was an important determinant of fluid strategy and it was shown to be statistically significant in our blip function. However, the mapping from (functional form of) mBP to fluid strategy was not similar in early versus later stage. On the first day, a parabolic function was fit with the turning point at 62.1 mmHg, which was very close to the 65 mmHg as recommended in the SSC guideline. At the later stages (Day 3 and 5), we found that previous urine output and treatment strategy were important determinants of current fluid strategy. This novel finding indicated that the optimal treatment strategy must take previous responses (e.g. urine output in response to fluid administration) into consideration.

Another strength of this study was that we calculated fluid strategy over 5 days after ICU admission. The majority of previous trials focused on the first 6 or 12 h to investigate the effect of fluid resuscitation strategy^1,21,22^. This may lead to unsatisfactory results of previous trials. We argued that fluid management should be carried out during the entire disease course. Our DTR model correctly captured the clinical variables over the dynamic process of sepsis and provide a sequential decision rules to maximize the survival time. Our result showed that the proportion of subjects being inappropriately treated with liberal fluid strategy increased from Day 1 to 5 (i.e. these patients can have longer survival time had they treated with restrictive fluid administration). More recently, the concept of de-resuscitation (active removal of fluid using diuretics or renal replacement therapy) after hemodynamic stabilization has received more and more attentions^26^. There was evidence that negative fluid balance achieved with de-resuscitative measures resulted in lower mortality^6^. These studies also highlighted the importance of careful fluid management in later phase after hemodynamic stabilization.

There are limitations in the current study. For example, the study population of sepsis was based on sepsis-2.0 definition, which may identify different population than that identified by using the most updated Sepsis-3.0 criteria^27^. However, the study was not a prospective study in which screening criteria could be prospectively collected. In the dataset, there was missing data on required items for the definition of sepsis-3.0. For example, the sepsis-3.0 definition requires an acute increase in the SOFA score, which means that we must have information for the baseline SOFA score to implement the Sepsis-3.0 definition. In fact, the database did not contain such complete information for the implementation of sepsis-3.0 criteria.

## Conclusions

In conclusion, the study successfully computed out a sequence of dynamic fluid management strategy for sepsis patients over the first 5 days after ICU admission with a large volume of electronic healthcare data. The decision rules on day 1, 3 and 5 adopted different functions of covariates and treatment histories. The optimal treatment strategy generated by the DTR model could significantly improve the survival outcome as compared with the actual fluid strategy. The decision rules developed in the study require further validation in prospective cohorts.

## Data Availability

Data were available on request.

## Abbreviations

SOFA: Sequential organ failure assessment
DTR: Dynamic treatment regimen
RL: Reinforcement learning
ICU: Intensive care unit
mBP: Mean blood pressure
HR: Heart rate
RR: Respiratory rate
CCU: Coronary artery unit
CSRU: Cardiac surgery recovery unit
MICU: Medical ICU
SICU: Surgical ICU
TSICU: Trauma-neuro ICU
RRT: Renal replacement therapy
IQR: Interquartile range
